# Novel Cell-Ess ® supplement used as a feed or as an initial boost to CHO serum free media results in a significant increase in protein yield and production

**DOI:** 10.1016/j.csbj.2016.07.001

**Published:** 2016-08-03

**Authors:** Adam Elhofy

**Affiliations:** Essential Pharmaceuticals, Ewing, New Jersey, United States

**Keywords:** Bioprocessing, Titer, Lipid, Cell-Ess, CHO, Monoclonal antibody, Serum free media, Animal component free, Viable cell density, Bioreactor, Single use bioreactor

## Abstract

Many metrics, including metabolic profiles, have been used to analyze cell health and optimize productivity. In this study, we investigated the ability of a lipid supplement to increase protein yield. At a concentration of 1% (v/v) the lipid supplement caused a significant increase in protein titer (1118 ± 65.4 ng 10^5^ cells^− 1^ days^− 1^) when compared to cultures grown in the absence of supplementation (819.3 ± 38.1 ng 10^5^ cells^− 1^ days^− 1^; p < 0.05). This equated to a 37% increase in productivity. Furthermore, metabolic profiles of ammonia, glutamate, lactate, and glucose were not significantly altered by the polar lipid supplement. In a separate set of experiments, using the supplement as a feed resulted in 2 notable effects. The first was a 25% increase in protein titer. The second was an extension of peak protein production from 1 day to 2 days. These results suggest that lipid supplementation is a promising avenue for enhancing protein production. In addition, our results also suggest that an increase in protein production may not necessarily require a change in the metabolic state of the cells.

## 1 Introduction

Using proteins as therapies was ushered in during the early 1980's with the introduction of EPO. Since then, the biologics market has seen a significant increase in the number of products and the total revenue generated each year. The sales of biologics have surpassed $125 billion [1]. As the market has grown, there has been increased competition as numerous companies target therapeutics for the same indication as well as new indications. In addition, biosimilars, already popular in Europe, are growing rapidly in the United States and elsewhere. This creates competitive price pressure and the need for biotech companies to optimize their protein production processes.

Enhancing protein production of cultured cells is a common goal throughout the biomanufacturing industry. This commonly consists of an ongoing process of optimizing cell lines in serum free conditions in order to balance cell proliferation and protein production.

Serum free media have been optimized based on certain principles. The original serum free media was made by Ham in the 1960s [2]. It was based on adding the amino acids, vitamins, and salts needed for cells to grow. The same concept is generally used as a base media today but as technology has improved so have the methods of analysis. Assays testing metabolic byproducts and investigating proteomics are used today to better optimize a media for CHO cell lines [3], [4], [5], [6], [7]. The use rate of amino acids or glucose can be determined to identify the constituents rapidly used during a run and may need replenishment, usually in the form of a feed and or feeds. None of these assays screen for lipids or cholesterol. In fact, the typical metabolic analysis looking at the TCA cycle would not be able to predict a benefit or show the harm of not having lipids and cholesterol. It has been reported that essential fats can be beneficial for cell health and protein production [6], [8], [9], [10].

Here we investigate the utility of a novel approach to optimization using Cell-Ess® as a feed or an initial supplement to show proof of principle of adding lipids to culture. Furthermore Cell-Ess is tested as a feed supplement and in a small bioreactor as a model for use in large scale bio-production and use in a single use bioreactor. In this report we hypothesize that adding lipids will benefit protein production and then set out a set of experiments to test the hypothesis.

## 2 Materials and methods

### 2.1 Cell culture

Suspension Chinese Hamster Ovary (CHO) cells expressing several different monoclonal antibodies (mAb) from multiple individual clones were grown in 50 mL suspension cultures for 7 days. The lipid supplement was administered using a dosing paradigm as indicated for each experiment. The effect on protein titer and common metabolic profiles were determined and analyzed.

For these experiments two different stably transfected mAb single isolated CHO cell clones were used. The CHO cells were either grown in SAFC (Sigma, St. Louis, MO) serum free media or FortiCHO (Thermo Life). CHO cells were thawed and expanded prior to seeding in shake flasks.

### 2.2 Supplement with Cell-Ess

Cells were seeded in shake flasks. Cell-Ess was given as a supplement independent of other supplements and in addition to normal feeds at increasing concentrations. Cell-Ess (Essential Pharma, Ewing, NJ) was either added at the beginning of culture for protein expression or added as a feed as indicated.

### 2.3 Protein analysis

The mAb titers were calculated using two different methods. For the GS0 cells grown in SAFC media the cells were purified on a protein A column and then quantified by in a spectrophotometer at a wavelength of 280. The mAb from the DG44 cells grown in FortiCHO were quantified by an antibody specific ELISA.

### 2.4 Metabolic analysis

Samples were taken at the indicated time points and frozen. At the end of the run the samples were thawed and run on the BioProfile Flex analyzer (Nova Biomedical, Waltham, MA).

## 3 Results

### 3.1 Addition of Cell-Ess® as a supplement significantly increases titer

Large-scale protein production has predominantly moved to serum free systems developed for CHO cell lines. Serum free media systems are made by multiple companies in the life science industry, like Thermo Fisher Scientific, one of the industry leaders. Thermo provides several brands of CHO cell media including but not limited to FortiCHO and OptiCHO. In theory the complete media systems are ready for use. Interestingly, even though there are readily available products, many biomanufacturers will make internal modifications to optimize protein production for a specific cell line suggesting genetic differences were introduced to each CHO cell clone. In this study we investigated if the addition of a lipid supplement, Cell-Ess®, would lead to an increase in protein production across different clones in two different genetic backgrounds. A multi-step approach was utilized where proof of principle was researched to test the hypothesis of the benefit of adding lipids in a simplified controlled model. The second step was to test a complex model for fed batch in shake flasks where there are multiple variables. The last part of the investigation was the addition of lipids to a scalable small bioreactor utilizing a single use bioreactor (SUB) where lipid additives previously.

The first study on proof of principle was designed to be open-ended allowing for a variety of outcomes. Four theoretical outcomes were possible; an advantage of using the lipid supplement at a defined percentage; an advantage of using Cell-Ess regardless of percentage of supplement used; the addition of the lipid supplement provided no effect; and lastly Cell-Ess ® would have a negative effect on protein titer.

**Fig. 1:**
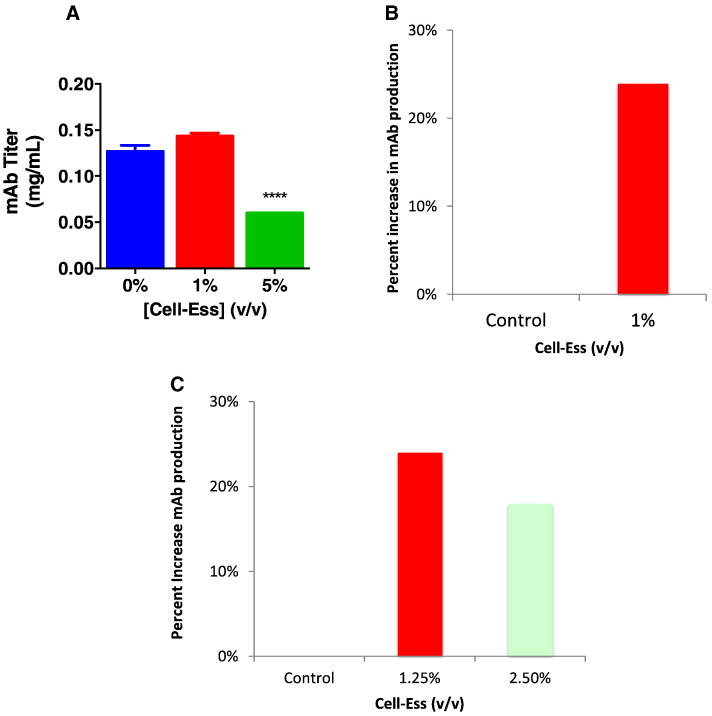
Increased mAb titer with initial lipid supplement. The addition of Cell-Ess as an initial supplement in a short 7 day culture model was done in multiple media systems investigating the ability of the lipids supplement to increase mAb titers over control. In this figure the increase in titer due to the addition of Cell-Ess to SAFC media is shown in 1A and in 1B the increase is shown as a percent over control. In a similar experiment with addition of lipid at a different site with a different CHO clone the cells were grown in the Thermo media, Forti-CHO and shown as a percent increase over control.

**Fig. 2:**
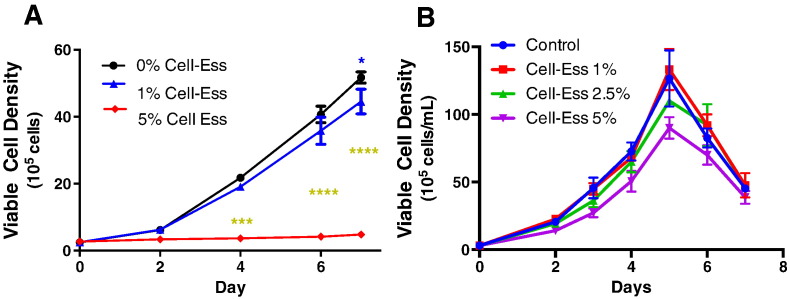
Viable cell density in mAb producing CHO cells with lipid supplement. Cell-Ess was added at increasing amounts in two independent experiments done with two different stably transfected CHO cell clones. In SAFC SFM (A) 5% Cell-Ess reduced growth while in FortiCHO media (B) 5% Cell-Ess had a similar growth pattern to control.

**Fig. 3:**
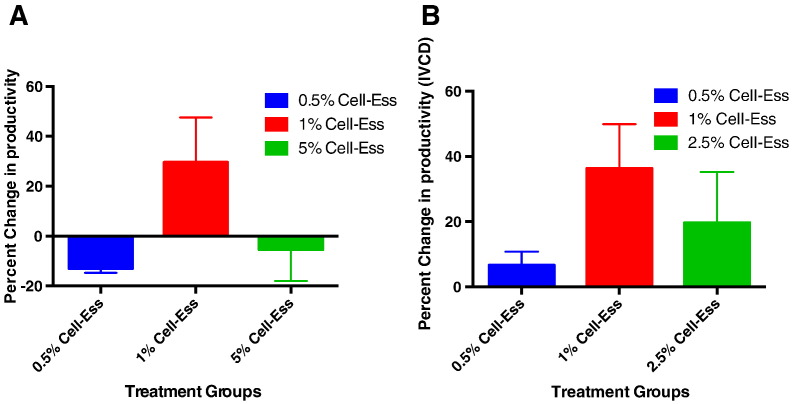
Increased CHO cell productivity with initial lipid supplement. The addition of Cell-Ess as an initial supplement in a short 7 day culture model was analyzed for the ability to increase productivity of CHO cells. In this figure the increase in productivity in SAFC media is measured by two different methods. In (A) the calculation is the final titer in g/l divided by the final VCD cells/l resulting in g/cell and then increase is shown as a percentage. In (B) the integral under the VCD curve is calculated and used as the denominator to divide into the final titer and shown as a percent increase over control.

**Fig. 4:**
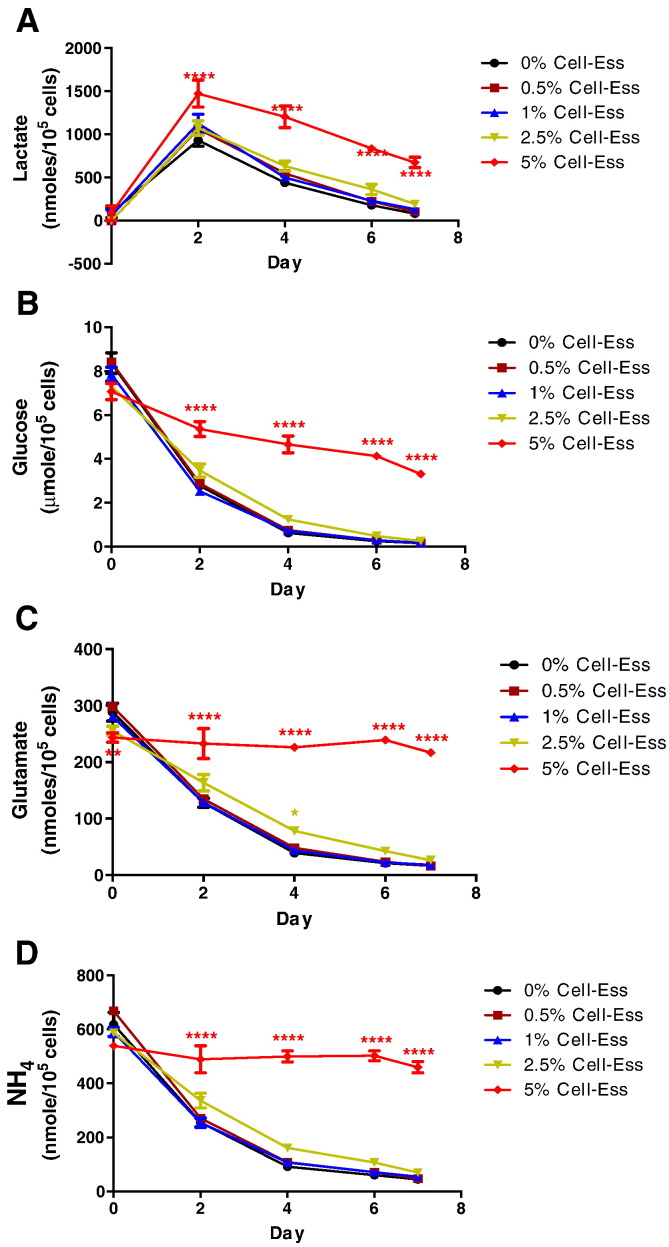
Addition of lipids does not affect the metabolic profile. Cell-Ess was added at the indicated levels to SAFC media at the indicated concentrations. Samples were collected from the associated flasks and frozen until the final collection. Samples were thawed and metabolic analysis for lactate, glucose, glutamate, and ammonia (A, B, C, and D respectively) was done using a BioProfile Nova Flex.

As mentioned previously, there are multiple manufacturers of serum free media where variability in performance has been observed. In an effort to understand how Cell-Ess ® can affect titer, we measured Cell-Ess's effect on titer output in two independent systems. One system utilized a clone designed for use specifically with FortiCHO and another clone was designed to produce specifically in Sigma's media. In a shake flask study the lipid supplement was added once at the initiation of culture. A range of Cell-Ess® was added to the shake flasks starting at 0.5%, up to 15%. We found there was a significant increase in monoclonal antibody production with the addition of 1% Cell-Ess ® (see Fig. 1). There was a statistically significant increase in titer at 1%, increasing output from 126 μgrams/ml to 143 μgrams/ml, a 25% increase using the SAFC media (see 1a and 1b). Similarly, the addition of the lipids to LifeTech's FortiCHO media(Fig. 1C) resulted in a similar and significant increase in titer. Interestingly, there was a significant increase with both the addition of 1% and 2.5% Cell-Ess. (See Fig. 2, Fig. 3, Fig. 4.)

### 3.2 Viable cell density is not altered by the addition of Cell-Ess ®

An increase in titer can be attributed to either an increase in cell density, an increase in the number of cells making the same amount of protein per cell, or increased protein production on a per cell basis. To better understand if the increase in titer was a result of increased cell density we investigated the viable cell density (VCD) and viability of the two different clones treated with Cell-Ess ®. At 0.5% and 1% additions of Cell-Ess, there was no alteration in VCD. At increasing amounts of Cell-Ess®, the VCD began to display an effect starting at the 2.5% concentration in SAFC media. In the Thermo Fisher media the VCD was also unchanged at 1% Cell-Ess supplementation but with the addition of 2.5% Cell-Ess there was no effect of the VCD. Adding Cell-Ess in a specified ranged of 1%–2.5% did not have any effect on proliferation or cell viability when added as an initial supplement. Since Cell-Ess did not increase the cell density the increase in titer was due to increased productivity, and not by more cells making the same or slightly less protein as in the perfusion model. This is often the case in constant perfusion systems.

### 3.3 There is a significant increase in the yield per cell using an initial supplement of Cell-Ess ®

There are multiple ways to measure protein output. One way is to measure titer where the addition of Cell-Ess increased titer. Another measure of output is productivity. In this assay we measured if there was an increase in the amount of productivity on a per cell basis. Productivity can be measured by one of several different methods. The most common methodology is to divide the titer from a set time point and then divide this by the number of cells at that same time point. The result is an indication of the productivity or amount of protein secreted on a per cell basis. The addition of Cell-Ess resulted in a significant increase in the amount of protein secreted per cell. This increase in productivity was not dependent on cell proliferation as there was no significant increase in total cell growth (129.2 ± 9.0 10^5^ cells day) when compared to control (145.2 ± 5.3 10^5^ cells day). At 1% addition of Cell-Ess, there was a greater than 40% increase in productivity. The addition of lipids increased the amount of total mAb titer by increasing the productivity of CHO cells. Another more conservative measure of productivity is to divide the titer of a single time point by the area under the VCD curve or IVCD. That value describes the production of the aggregate production over the entire time frame. The addition of Cell-Ess increased productivity of cells using the IVCD method regardless of Cell-Ess concentration. These data suggest the addition of lipids allows CHO cells to more efficiently produce protein.

### 3.4 There is no alteration in the metabolites or salts despite a significant increase in mAb production

Since the addition of lipids increases titer primarily by increasing the efficiency of CHO cells, an experiment was then conducted to determine if the increased protein secretion was causing the CHO cells to build up toxic by- products. As mentioned in the introduction, metabolic analysis has been used to optimize CHO cell antibody production. We examined the entire metabolic profile (Fig. 3) and identified no significant differences in any of the salts or metabolic by-products. These are common indicators for potential harmful by-products. Lactate is one factor that can increase the pH causing cell necrosis. In no fewer than 6 independent runs in both models, addition of Cell-Ess as an initial supplement or as a fed batch supplement did not result in an accumulation of lactate. In fact after the initial peak, lactate was controlled while simultaneously there was a constant demand for glucose. The addition of lipids did not feed into the TCA cycle which would result in an increase in lactate as occurs with the addition of glucose. Since there was an increase in the amount of protein, there is a potential for the increase the amount of ammonia, a toxic by-product of additional protein.

### 3.5 The addition of lipids in a fed batch system results in increased titers

The most common method to extend culture times and increase titer is to use a feed during the batch run. The method, called fed batch, uses a variety of feeds to add back nutrients which were utilized by the cells in the bioreactor. There is a way to model the fed batch system in shake flasks. This study sought to understand - in a system where feeds are added to an optimized serum free culture condition - if the use of Cell-Ess as a feed can increase titer or have other beneficial effects, such as improving cell viability or extending potential run times.

**Fig. 5:**
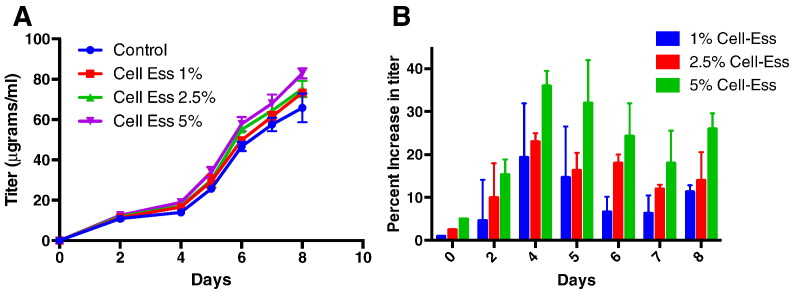
Increased mAb titer with a lipid as feed in a fed batch model. Cell-Ess was used as an initial supplement and as a feed in shake flask model for fed-batch protein production. Cell-Ess was added as an initial supplement at 1% as determined in Fig. 1 and then increasing feed amounts were added as indicated. Protein was quantified by ELISA shown in A and the increase in titer as a percent is shown in B.

To test this hypothesis we modeled a fed batch system in shake flasks, with the study variable being feeds of Cell-Ess® at varying concentrations. Feeds already utilized in these previously optimized systems were retained to control for the effect of adding Cell-Ess. There was a benefit of Cell-Ess at all concentrations tested in the fed batch model unlike the use of the lipid supplement Cell-Ess when used as a one-time initial supplement. The optimal result was seen with multiple feedings of Cell-Ess at a concentration of 5%. As seen in Fig. 5, there were multiple benefits. The first is an increase in titer over the controls. The increase in titer was greater than 30%. The second observation was the addition of Cell-Ess led to an increase in titer early, and throughout the run. Finally, the viability improved with the use of Cell-Ess. The addition of lipids as a feed behaved similarly to when the lipids were used only as an initial supplement. The titer was significantly increased without increasing the cell proliferation. In both models there was a productivity increase through the use of Cell-Ess.

### 3.6 A novel liposome can be used in a single use bioreactor

Shake flasks experiments are very useful in modeling behavior and optimizing conditions prior to entering a bioreactor. The behavior of the culture in bioreactors can be different than that seen in shake flasks. We then wanted to determine if the lipid supplement, could be used to increase titer as shown in the shake flask model Fig. 1, Fig. 5, would be repeatable in a bioreactor. This experiment involved testing Cell-Ess as both an initial supplement at 1% and also adding it as a feed at 5% as determined in the shake flask model (Fig. 5). Furthermore, we wanted to test the lipid supplement in a single use system where lipid use has been shown to be toxic or not beneficial.

Cell-Ess was tested in a 10 l Wave bag with EVA film. Bioreactor runs done at the 5 L size have been shown to be representative for large-scale bioreactor runs up to the 10,000 L scale. The hypothesis would be the use of Cell-Ess in a SUB would be as efficacious as the run done in the smaller volume shake flasks.

## 4 Discussion

The use of lipids has been shown to be beneficial but to date there has not been an optimized method to deliver the lipids into a long term culture system. Long term culture systems are the standard for growing and expanding CHO cells for protein production. Many methods have been employed to determine the optimal media for each CHO cell and CHO cell clone [7], [11], [12]. The methods include, but are not limited to, metabolic analysis [3], [11], [12], proteomic analysis [11], [13], and network analysis. In this paper we demonstrated a novel lipid mixture using a proprietary delivery system called Cell-Ess® can deliver lipids and cholesterol resulting in increased titer by increasing cell productivity.

During the process of empirically testing Cell-Ess, several interesting discoveries were made related to supplementing lipids in a serum free environment. It was found during the proof of principle experiment, where Cell-Ess was only added at the beginning of a short protein run, the amount of lipids might have different effects between different media When 1% Cell-Ess was added to FortiCHO and SAFC media there was an equivalent increase in mAb titer. But the FortiCHO SFM supported growth and increased titer with concentration of lipids greater than 1% while with more than 1% Cell-Ess in SAFC media did not sustain growth, demonstrating there was a different sensitivity based on constituents in the SFM. There was an effect on the proliferation of CHO cells at 2.5% in the SAFC media but, at 2.5% the FortiCHO did not change the proliferation and resulted in increased titer. We found this effect was much broader in the fed batch experiment. In data not shown here, the number of feeds and the Cell-Ess® concentration per feed varied greatly between media but the result - once optimized - was the same. There was significant increase in the titer in both models. It is critical to optimize the addition of lipids. While we often assume the difference in formulations explain why there are different optimization parameters, it is not clear what specific ingredient or ingredients are contributing to this variance.

There is a movement toward single use systems [11], [14]. The single use systems take advantage of a smaller bioreactor and utilize a constant perfusion to replenish the media and maintain cell health. The result is increased cell density and increased titer. The increase in cell density is in the range of 3–5 fold while the increase in titer is usually 1.5 to 2 fold. From a downstream processing perspective, this approach increases the amount of cell bioburden and host cell protein on a per gram purification. An increase in productivity (seen in figure 2) is more desirable as the amount of protein per cell is increased while the purification burden downstream is not affected. The use of the novel lipid supplement Cell-Ess® increases the productivity on a per cell basis. Furthermore Cell-Ess functioned equally well in glass and on a single use film.

**Fig. 6:**
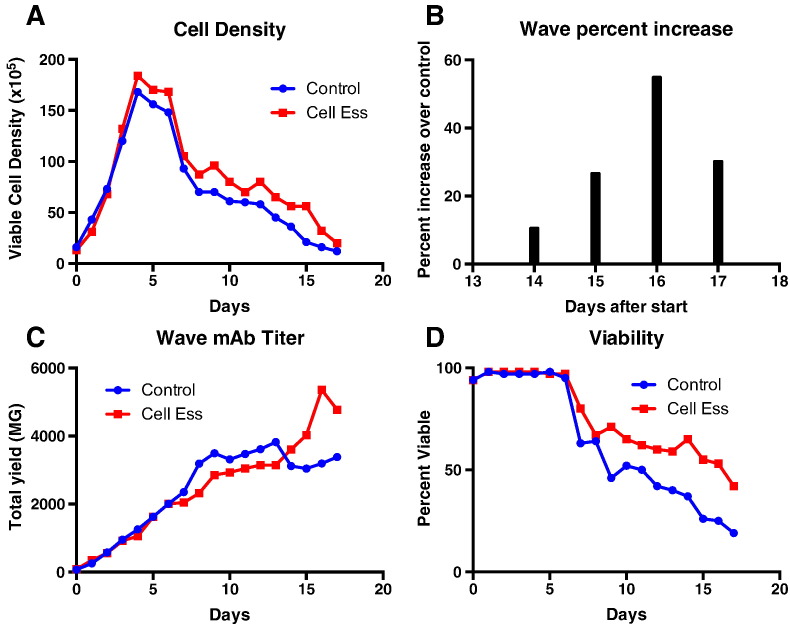
Increased mAb titer in single use bioreactor. Cell-Ess was used as an initial supplement and as a feed in a single use bioreactor (SUB). A 5 l batch run was done in 10 l Wave bags. Cell-Ess was added as an initial supplement at 1% as determined in Fig. 1 and then 5% feed was added every 3 days. Protein was quantified by ELISA shown in C and the increase in titer as a percent is shown in B. The VCD and viability rates are shown in A and D respectively.

There are numerous supplements on the market provided by a variety of companies, but in many cases the supplement is designed to work with a specific media formulation. Cell-Ess is designed to be robust so the results are media agnostic. To date, Cell-Ess has been added to SAFC, LifeTech (Fig. 1, Fig. 5, Fig. 6) and Lonza media (data not shown) with an increase in titer observed all systems. This was done at difference sites with different clones and reproduced multiple times with the same result. As mentioned earlier, the design of an experiment is crucial to define the optimal amount of lipid supplement for establishing reproducible results.

The methods used to optimize CHO cell production, such as metabolic analysis, and proteomic analysis will not predict or demonstrate the benefit of adding lipids. The output is focused on the TCA cycle or what proteins are being produced. Here it was demonstrated that there were no alterations in the metabolic profile even while there was a significant increase in the protein titer. This suggests the increase in titer does not alter the TCA cycle since there is no increased lactate. This can mean there is more efficient energy usage which the cell can then utilize for increased protein production. There have been reports recently suggesting a metabolic profile in the oxidative state is conducive to increased titer [3], [4]. Another explanation is the lipid's stabilizing effect on the lipid membranes throughout the entire protein synthesis and secretion pathway.

This study showed the addition of lipids increased mAb titer by increasing productivity. This increase did not affect the metabolic profile of the cell. The next level of investigation will be on protein quality. While preliminary data does exist that is not shown here, the glycolytic pattern is not significantly altered by the addition of Cell-Ess. Further exploration of the role of adding lipids on protein quality is planned.

## Conflicts

I am employed by Essential Pharmaceuticals, LLC the maker of Cell-Ess. The work done in the manuscript was done by third parties who did not have any financial interest in Essential Pharmaceuticals, LLC or receive any incentives based on the outcome of experiments done.
